# Supplementary material for: Oxidative Ferritin Destruction: A Key Mechanism of Iron Overload in Acetaminophen-Induced Hepatocyte Ferroptosis
Source: Int J Mol Sci. 2025 Aug 5;26(15):7585. doi: 10.3390/ijms26157585 (PMC12347875; doi:10.3390/ijms26157585)
Supplement: Supplementary file 1 [file ijms-26-07585-s001.zip › ijms-3777503-supplementary.pdf]

Supplementary Materials

1. Morphological changes in primary mouse hepatocytes following APAP exposure.

After exposure to 10–30 mM APAP, the hepatocytes maintained normal adherence with intact cellular architecture, distinct cellular boundaries, and preservation of nuclear morphology in both mononuclear and binuclear populations. Progressive morphological changes became evident at 4 hours post-treatment. APAP with concentrations more than 10 mM induced extensive cytoplasmic vacuolization, higher concentrations (20–30 mM) triggered cellular shrinkage and partial membrane disruption. After 6 hours' exposure, PMHs showed gradual loss of nuclear membrane definition across all concentration groups. The 20−30 mM treatment groups exhibited complete nuclear dissolution, marked cellular shrinkage, cytoplasmic reduction, and loss of cellular demarcation.

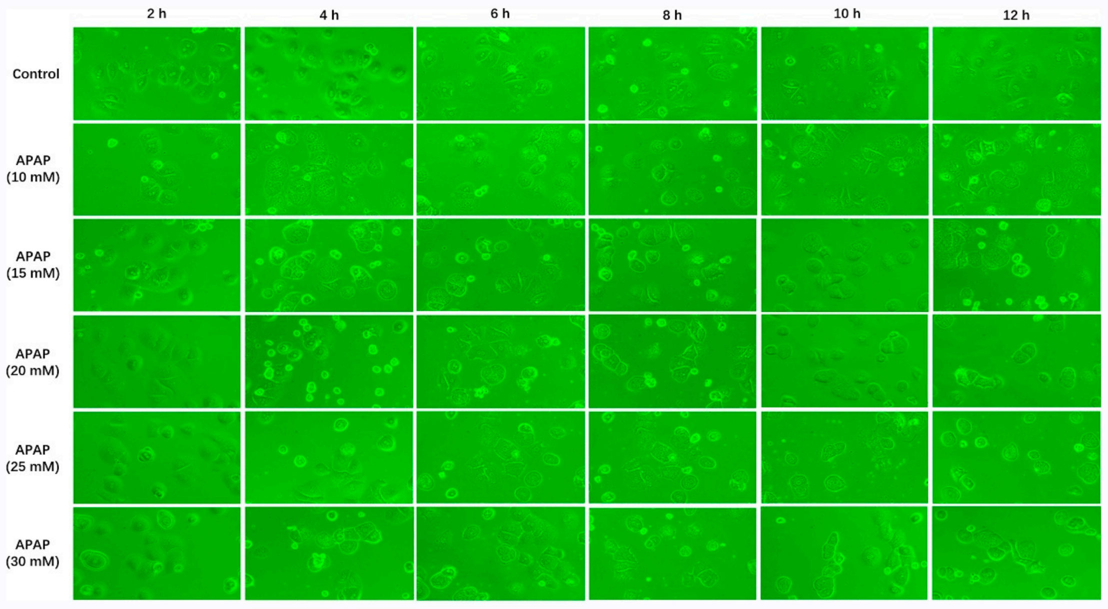

Figure S1. Cells morphology changes induced by APAP after treatment for 2–12 h.

2. Co-localization of ferritin and lysosomes

Immunofluorescence was performed to determine the co-localization of ferritin and lysosome. Briefly, freshly isolated PMHs were seeded onto 35 mm glass-bottom dishes at a density of 3×10^5 cells/well in 2 mL DMEM/H medium and allowed to adhere for 12 hours. Cells were then divided into solvent control (0.67% DMSO), model (20 mM APAP), and treatment groups (2 mM NAC, 2 mM DFO, or 400 μM CRO) and incubated for an additional 12 hours. Following treatment, cells were washed three times with PBS and stained with 50 nM LysoTracker Green in the dark at room temperature for 1 hour. After three PBS washes, cells were fixed with 4% paraformaldehyde (800 μL/well) for 20 minutes, then permeabilized with 0.5% Triton X-100 for 10 minutes, and blocked with 2% BSA for 1 hour at room temperature. Cells were then incubated with anti-FTH1 primary antibody (1:2000 dilution) at 4°C overnight. After washing with PBS, the cells were incubated with Cy3-conjugated secondary antibody for 1 hour in the dark. Nuclei were counterstained with DAPI (1:100) for 5 minutes. Samples were then imaged using confocal microscopy.

The results showed that lysosomes exhibited uniform cytoplasmic distribution with high fluorescence intensity, while ferritin demonstrated both cytoplasmic and nuclear localization, with predominant nuclear accumulation in control PMHs (Figure S2). APAP treatment induced significant reduction in lysosomal fluorescence intensity and appearance of cytoplasmic

vesicular structures containing aggregated green fluorescence. In APAP treatment PMHs, partial cytoplasmic ferritin depletion was observed with minimal formation of red fluorescent aggregates. These ferritin aggregates showed precise colocalization with lysosomal clusters. DFO and NAC treatment partially restored cytoplasmic ferritin fluorescence intensity without altering the colocalization pattern. In contrast, CRO treatment showed no significant effects on either ferritin distribution or ferritin-lysosome colocalization.

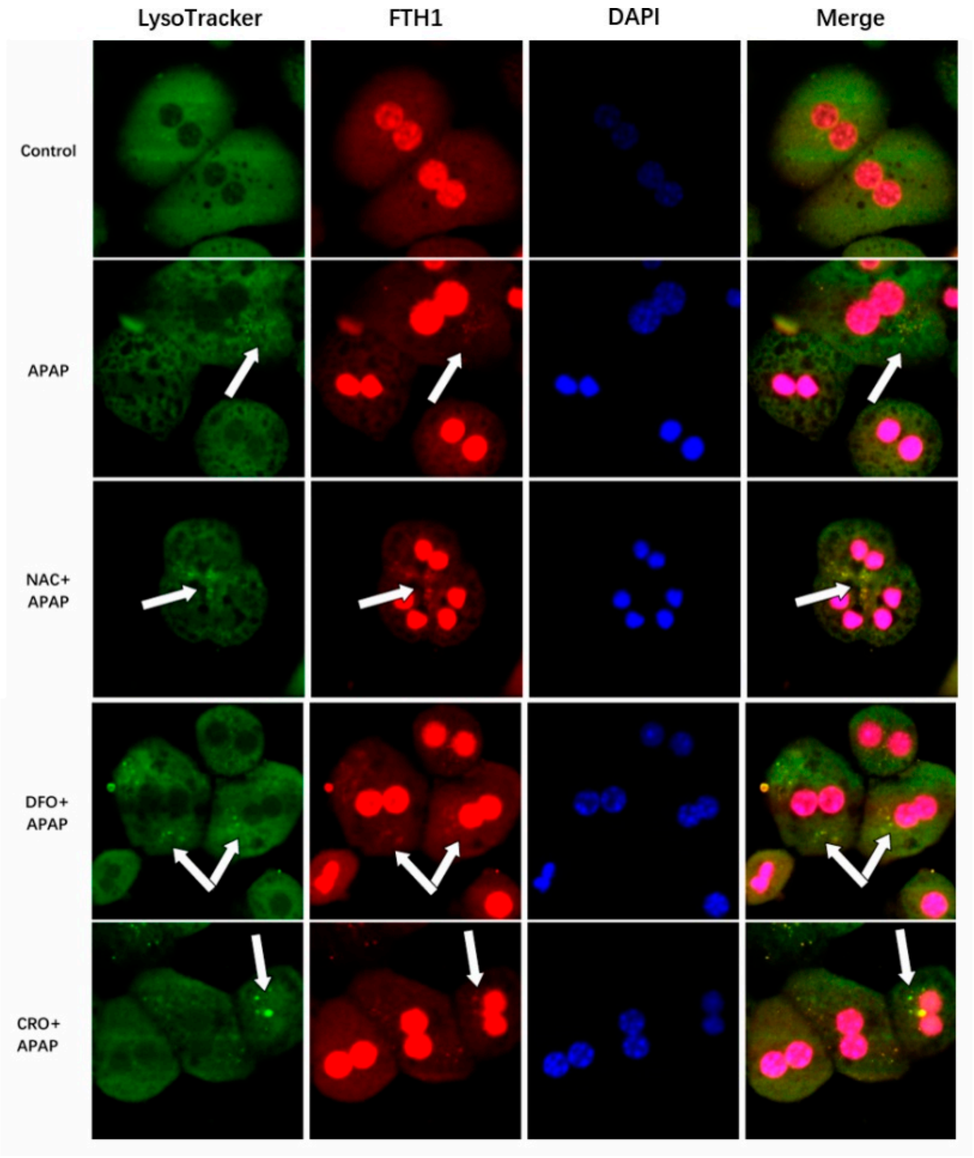

Figure S2. Lysosome-ferritin co-localization analysis in APAP-treated hepatocytes (× 1000). Confocal microscopy revealed distinct staining colors of lysosomes (green), ferritin (red), and nuclei (blue). Control hepatocytes showed uniform lysosomal distribution with high fluorescence intensity throughout the cytoplasm, while ferritin showed predominant nuclear localization and faint cytoplasmic staining. APAP treatment induced significant lysosomal depletion (reduced green fluorescence) accompanied by cytoplasmic vacuolization. Sparse lysosomal (white arrow) and ferritin (white arrow) aggregates demonstrated minimal co-localization. Both DFO and NAC treatment partially restored cytoplasmic ferritin fluorescence intensity without affecting colocalization, while CRO showed no significant effects.

3. The standard curve and the presentative chromatogram.

A series calibration working standards of GSH 1, 10, 100, 500, 1000 μM were prepared. The regression equation of the calibration curve was y=7.6174x+10.453 with R^2=1, where y is the peak area count of UV absorbance and x is the concentration (μM) of GSH standard solutions (Figure S3 A). Typical chromatograms of blank, standard and sample were depicted in Figure S3 B.

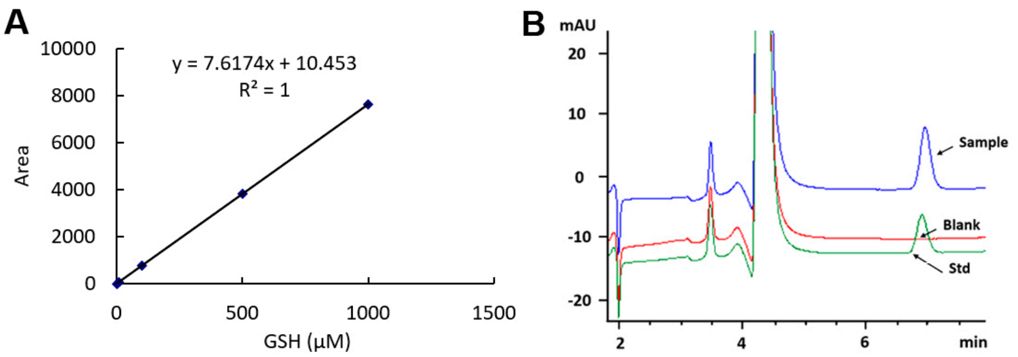

Figure S3. The standard curve (A) and the presentative chromatogram for blank, standard, and sample (B).
